# [Corrigendum] Astragaloside IV alleviates heart failure by regulating SUMO‑specific protease 1

**DOI:** 10.3892/etm.2023.12021

**Published:** 2023-05-16

**Authors:** Juan Liu, Ya Li, Xiyun Bian, Na Xue, Jiancai Yu, Shipeng Dai, Xiaozhi Liu

Exp Ther Med 22:1076, 2021; DOI: 10.3892/etm.2021.10510

Following the publication of the above article, an interested reader drew to the authors' attention that, in Fig. 7A on p. 7, they had inadvertently uploaded an incorrect image for the ‘HA-SENP1/ISO/Monomer’ data panel, which had appeared in one of this research group’s previous publications [Bian X, Xu J, Zhao H, Zheng Q, Xiao X, Ma X, Li Y and Liu X: Zinc-Induced SUMOylation of Dynamin-Related Protein 1 Protects the Heart against Ischemia-Reperfusion Injury. Oxid Med Cell Longev 22: 1232146, 2019].

The authors have re-examined their original data, and realize which data panel should have been included for this experiment in this figure. The corrected version of Fig. 7, now showing the correct data for the HA-SENP1/ISO experiment, is shown below. Note that the error made in assembling this figure incorrectly did not affect the overall conclusions reported in the paper. All the authors agree with the publication of this corrigendum, and are grateful to the Editor of *Experimental and Therapeutic Medicine* for allowing them the opportunity to publish this. They also apologize to the readership for any inconvenience caused.

## Figures and Tables

**Figure 7:**
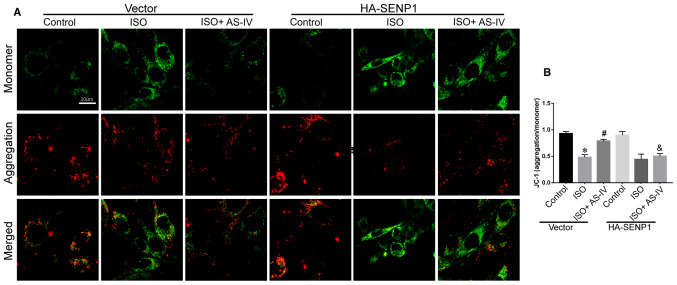
Effect of Senp1-overexpression on mitochondrial membrane potential in HL-1 cells. (A) Compared with the control (dimethyl sulfoxide, 0.1%), the JC-1 ratio (aggregate/monomer) was decreased in 20 µmol/l ISO-induced HL-1 cells. AS-IV (50 µmol/l, n=7) prevented the ISO-induced decrease in the JC-1 ratio, which was inhibited by Senp1-overexpression (n=8). (B) Summarized data of the JC-1 ratio. Data are presented as the mean ± standard deviation. Two-way analysis of variance followed by Bonferroni’s test. *P<0.05 vs. control; #P<0.05 vs. HF; &P<0.05 vs. HF+AS-IV. Senp1, small ubiquitin-like modifier-specific protease 1; ISO, isoprenaline; AS-IV, astragaloside.

